# The Effects of Neuropeptide B on Proliferation and Differentiation of Porcine White Preadipocytes into Mature Adipocytes

**DOI:** 10.3390/ijms24076096

**Published:** 2023-03-23

**Authors:** Tatiana Wojciechowicz, Paweł A. Kolodziejski, Maria Billert, Mathias Z. Strowski, Krzysztof W. Nowak, Marek Skrzypski

**Affiliations:** 1 Department of Animal Physiology, Biochemistry and Biostructure, Poznan University of Life Sciences, 60-637 Poznan, Poland; 2 Department of Hepatology and Gastroenterology, Charité-University Medicine Berlin, 13353 Berlin, Germany; 3 Medical Clinic III, 15236 Frankfurt, Germany

**Keywords:** adipocytes, adipogenesis, differentiation, neuropeptide B, preadipocytes, proliferation, porcine, p38

## Abstract

Neuropeptide B (NPB) affects energy homeostasis and metabolism by binding and activating NPBWR1 and NPBWR2 in humans and pigs. Recently, we reported that NPB promotes the adipogenesis of rat white and brown preadipocytes as well as 3T3-L1 cells. In the present study, we evaluated the effects of NPB on the proliferation and differentiation of white porcine preadipocytes into mature adipocytes. We identified the presence of NPB, NPBWR1, and NPBWR2 on the mRNA and protein levels in porcine white preadipocytes. During the differentiation process, NPB increased the mRNA expression of PPARγ, C/EBPβ, C/EBPα, PPARγ, and C/EBPβ protein production in porcine preadipocytes. Furthermore, NPB stimulated lipid accumulation in porcine preadipocytes. Moreover, NPB promoted the phosphorylation of the p38 kinase in porcine preadipocytes, but failed to induce ERK1/2 phosphorylation. NPB failed to stimulate the expression of C/EBPβ in the presence of the p38 inhibitor. Taken together, we report that NPB promotes the differentiation of porcine preadipocytes via a p38-dependent mechanism.

## 1. Introduction

Neuropeptide B (NPB) is a peptide hormone identified in 2002. NPB is post-translationally modified by bromination of the N-terminal tryptophan residue, and composed of 23 (NPB-23) or 29 (NPB-29, C-terminally extended form) amino acids, respectively [1,2,3]. This unique bromine modification does not affect the binding affinity of NPB to its receptor. In humans, biological effects of NPB are mediated by the activation of NPBWR1 and NPBWR2 receptors [3]. Both receptor isoforms, initially named GPR7 and GPR8, share a 70% nucleotide and 64% amino acid sequence identity [4]. Rodents only express NPBWR1, whereas NPBWR2 is absent [3]. In pigs, both NPBWR1 and NPBWR2 receptors were cloned and sequenced [5]. Fang et al. [5] demonstrated the widespread distribution of NPBWR1 and NPBWR2 mRNA in the central nervous system (such as medulla oblongata, midbrain, hypothalamus, cerebellum, cerebral cortex, hippocampus, spinal cord) as well as in peripheral tissues (e.g., stomach, liver, pancreas, heart, bowels, spleen, ovary, testis, uterus, fat tissue, thyroid gland, kidney). NPB and/or its corresponding receptors were identified in other animal species such as pigs [6], chickens [7], or medaka fish [8]. There is cumulative evidence that NPB as well as its receptors are involved in controlling energy homeostasis by interacting with the central nervous system and peripheral tissues. It was shown that intracerebroventricular injection of NPB (3 nmol) biphasically and dose-dependently modulates appetite; an initial stimulation of food intake during the first 2 h was followed by a suppression of food intake. In contrast, a high dose of NPB (10 nmol) only had an anorexic effect [3]. NPB also stimulates cortisol production from zona fasciculata-reticularis (ZF/R) cells of the human adrenal cortex [9] and increases insulin secretion in the rat INS-1E cell line [10]. Furthermore, NPB was implicated in controlling the reproductive system [11,12] and modulating pain sensation [13]. Recently, we provided evidence that NPB may be involved in controlling white and brown adipogenesis. Using rodent primary preadipocytes as well as the murine 3T3-L1 cell line, we found that NBP stimulates the proliferation and differentiation of brown and white preadipocytes [14]. These data suggest that NPB may be involved in controlling fat tissue formation in rodents.

Adipogenesis is a complex process of forming lipid-filled mature cells from precursor fibroblast-like preadipocytes [15]. Preadipocytes are a part of mesenchymal stem cells (MSCs), located in the stromal vascular fraction (SVF) that can be isolated from adipose fat pads. These precursor cells can differentiate into lipoblasts, preadipocytes, and finally into mature adipocytes. Adipogenesis is a multi-step process encompassing cascades of transcription factors for crucial proteins that induce gene expression to form mature adipocytes. It is well-known that the major factors of adipogenesis molecular cascade are: peroxisome proliferator-activated receptor gamma (PPARγ), CCAAT/enhancer-binding proteins (c/EBPs such as C/EBPα, β and δ), and sterol regulatory element binding protein (SREBP). Fatty acid binding protein 4 (FABP4, known also as adipocyte protein 2, aP2), adiponectin, and fatty acid synthase (FAS) are responsible for the formation of mature, lipid-filled adipocytes [16,17].

Growth arrest is required for the induction of the adipogenic program followed by the expression of specific mRNA and protein markers of the early, intermediate, and late stages of differentiation, finally leading to triglyceride accumulation and the formation of metabolically active mature adipocytes [15]. At the early stage of differentiation, activation of the transcription factors C/EBPβ and PPARγ induces the arrest of cell growth. In addition, during this step of adipogenesis, adipocyte-specific genes are transactivated. The next step is characterized by the induction of the expression of the C/EBPα isoform, which is considered as a marker of mid stage differentiation. C/EBPα protein transactivates the gene promoters of FABP4, glucose transporter type 4 (GLUT-4), phosphenolpyruvate carboxykinase (PEPCK), leptin, and the insulin receptor [18,19,20,21]. In the process of differentiation, cells reach a spherical morphology from initially fibroblastic, and start to produce extracellular matrix (ECM) elements [22]. In the later stage, also known as the terminal stage of adipogenesis, de novo lipogenesis strongly increases, and adipocytes become insulin sensitive [23,24,25]. The newly formed mature adipocytes maintain a high expression of PPARγ and C/EBPα.

There is evidence that the crucial stages of adipogenesis including the expression of the major transcription factors are mediated through the members of the family of mitogen activated protein kinases (MAPKs). MAPKs encompass three main pathways: ERK1/2-, JNK- and p38-dependent [26]. We previously demonstrated that NPB can potentiate the differentiation of white rodent preadipocytes into adipocytes via the p38-dependent mechanism [27]. The p38 kinases are involved in processing cellular responses to nearly all stress factors [28]. It was undoubtedly confirmed that the p38 kinase requires phosphorylation at both the Thr and Tyr sites in the activation loop to gain the full enzymatic activation and efficiently process signal transduction [29].

Nevertheless, it needs to be pointed out that numerous studies addressing the role of p38 in the differentiation process provided contradictory results. P38 was reported to promote the differentiation of preadipocytes into mature adipocytes [17,18]. However, a suppression of this process has also been reported [19,20].

However, a proteomic and phosphoproteomic analysis performed by Rabbie et al. showed that most putative protein kinases involved in early adipogenesis belong to the cyclin-dependent kinase (CDK) and MAPK family, which also includes p38 [30]. The results reported by Hata et al. suggest that p38 kinase signaling is concomitantly activated and responsible for BMP2-induced adipocytic differentiation by inducing and upregulating PPARγ [31]. Moreover, pharmacological blocking of p38 activity in 3T3-L1 cells attenuated the expression of adipogenic markers and genes involved in lipid metabolism in the adipocytes (adiponectin and lipoprotein lipase) in monocyte chemotactic protein-1-induced adipogenesis [32], providing evidence for the proadipogenic role of the p38 family.

Currently, factors and mechanisms modulating adipogenesis are extensively studied in the context of obesity, obesity-related metabolic abnormalities as well as fat tissue remodeling [33]. It is well-known that due to the physiological, anatomical, and nutritional similarities, one of the best animal models suitable for studying human physiology in health and diseases is the pig *Sus scrofa* [34].

Furthermore, to improve the economic porcine meat production as well as breeding progress, the identification of novel factors modulating fat tissue development and deposition is of crucial relevance [35]. Therefore, in the present study, we evaluated the effects of NPB on the proliferation and differentiation of porcine preadipocytes into mature adipocytes [15].

## 2. Results

### 2.1. NPB and Their Receptors Are Present in Porcine Preadipocytes

**Figure 1:**
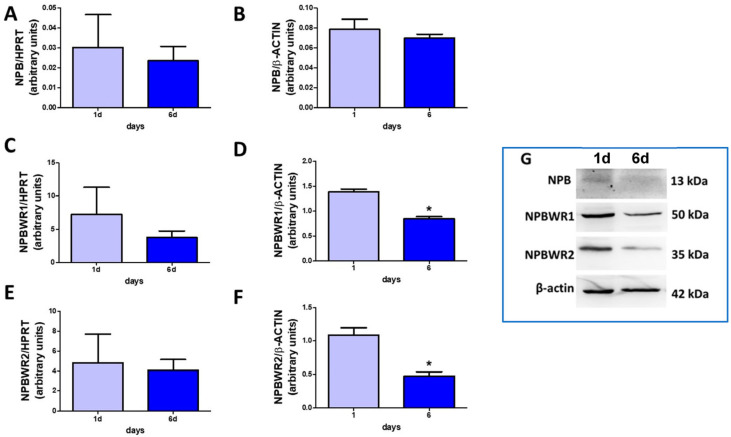
Expression of NPB, NPBWR1, and NPBWR2 in the porcine preadipocytes. Expression of NPB (**A**), NPBWR1 (**C**), and NPBWR2 (**E**) at the mRNA level in porcine preadipocytes differentiated for one or six days. Protein production of NPB (**B**), NPBWR1 (**D**), and NPBWR2 (**F**) in porcine preadipocytes differentiated for one or six days. Western blots represent protein production (**G**). Results are the mean ± SEM (n = 3–4). Statistical significance was considered when *p* < 0.05 (*).

As demonstrated in Figure 1, NPB mRNA (A) and protein (B) were present at comparable levels in cells differentiated for one and six days. Furthermore, we detected NPBWR1 and NPBWR2 in preadipocytes differentiated for one and six days at both the mRNA (Figure 1C,E) and protein levels (Figure 1D,F). Nevertheless, the protein production of NPBWR1 (Figure 1D) and NPBWR2 (Figure 1F) was lower in the cells differentiated for six days. These results indicate that NPB as well as both of its receptors are present in porcine preadipocytes.

### 2.2. NPB Promotes Proliferation of Porcine Preadipocytes

**Figure 2:**
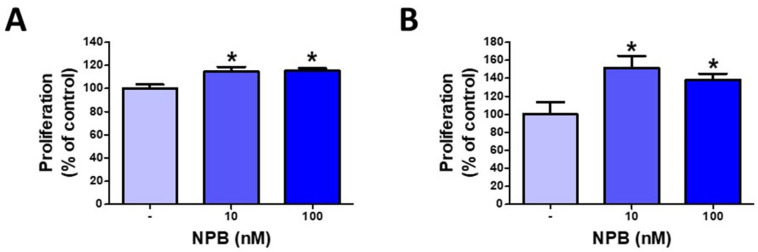
The effects of NPB on porcine preadipocyte proliferation. Effects of NPB (10 or 100 nmol/L) on proliferation in porcine preadipocytes assessed after 24 h (**A**) and 48 h (**B**). Results are the mean ± SEM (n = 5–7). Statistical significance was considered when *p* < 0.05 (*).

As shown in Figure 2, NPB (10 and 100 nmol/L) increased the proliferation of porcine white preadipocytes incubated for 24 (Figure 2A) and 48 h (Figure 2B).

### 2.3. NPB Stimulates Differentiation of Porcine Preadipocytes into Adipocytes

**Figure 3:**
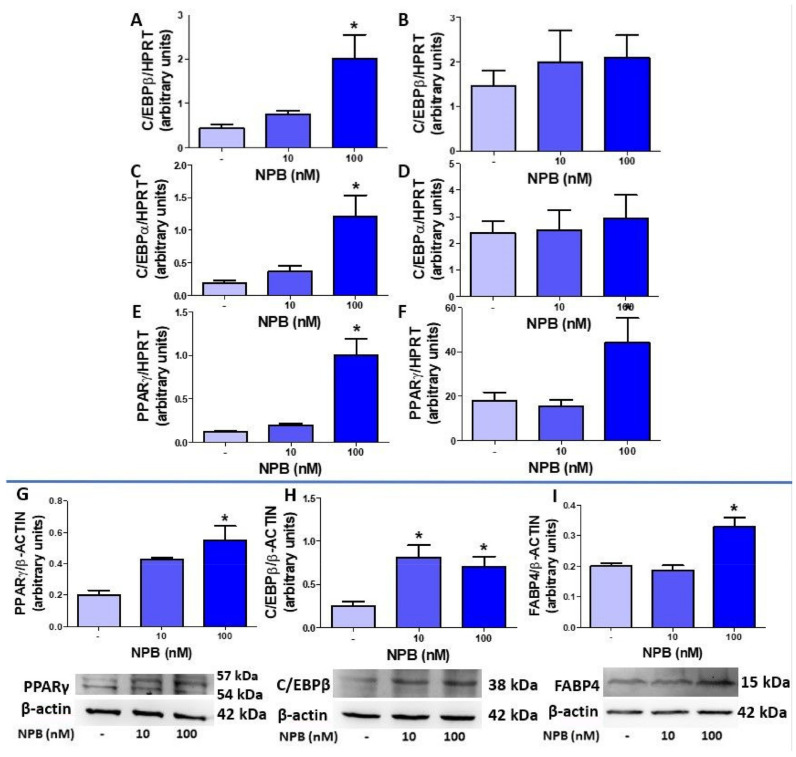
Effects of NPB on proadipogenic gene expression and protein production in porcine preadipocytes. Expression of C/EBPβ one (**A**) and six (**B**) days after the onset of differentiation, C/EBPα (day one—(**C**), day six—(**D**)) and PPARγ (day one—(**E**), day six—(**F**)) mRNA in cells differentiated with or without NPB (10 or 100 nmol/L) for one or six days. Effects of NPB (10 or 100 nmol/L) on PPARγ (**G**), C/EBPβ (**H**), and FABP4 (**I**) protein production assessed in porcine preadipocytes, differentiated for one day. Results are the mean ± SEM (n = 5–7). Statistical significance was considered when *p* < 0.05 (*).

To study the effects of NPB on adipogenesis, we investigated the effects of NPB on the expression of mRNA of the transcription factors and proteins involved in the differentiation of white preadipocytes into mature adipocytes. NPB (100 nmol/L) stimulated the expression of C/EBPβ (Figure 3A) and C/EBPα (Figure 3C) in cells differentiated for one day.

Next, we found that NPB (100 nmol/L) enhanced the expression of PPARγ in cells differentiated for one (Figure 3E) and six days (Figure 3F). In contrast, the expression of C/EBPβ and C/EBPα was not affected by NPB as assessed in cells differentiated for six days (Figure 3B,D). Moreover, preadipocytes differentiated in the presence of NPB (nmol/L) displayed an increased production of PPARγ (Figure 3G) and C/EBPβ proteins (Figure 3H). Furthermore, we detected that FABP4 protein production was increased in preadipocytes differentiated in the presence of 100 nmol/L NPB for one day only (Figure 3I).

**Figure 4:**
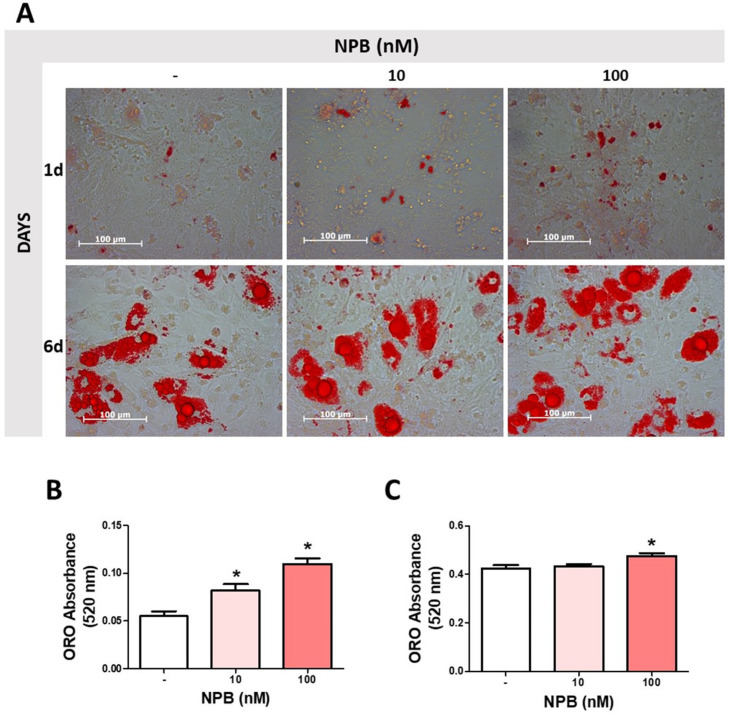
Effects of NPB on intracellular lipid accumulation in porcine preadipocytes. Representative images of rat preadipocytes differentiated with or without NPB (10 or 100 nmol/L) for one or six days (**A**). Effects of NPB (10 or 100 nmol/L) on the intracellular lipid content in porcine preadipocytes differentiated for one (**B**) or six days (**C**). Results are the mean ± SEM (n = 5–7). Statistical significance was considered when *p* < 0.05 (*).

Next, we assessed the influence of NPB on lipid accumulation during the differentiation process. As shown in Figure 4B, NPB at 10 and 100 nmol/L increased the lipid accumulation and lipid droplet formation in preadipocytes differentiated for one day. In addition, 100 nmol/L NPB increased the intracellular lipid content in cells differentiated for six days (Figure 4C). Microphotographs of WAT cell culture stained with ORO (400× magnification) are shown in Figure 4A.

Taken together, these results show that NPB promotes the differentiation of porcine preadipocytes into mature fat cells.

### 2.4. NPB Modulates Adipogenesis in Porcine Preadipocytes via p38-Dependent Mechanism

**Figure 5:**
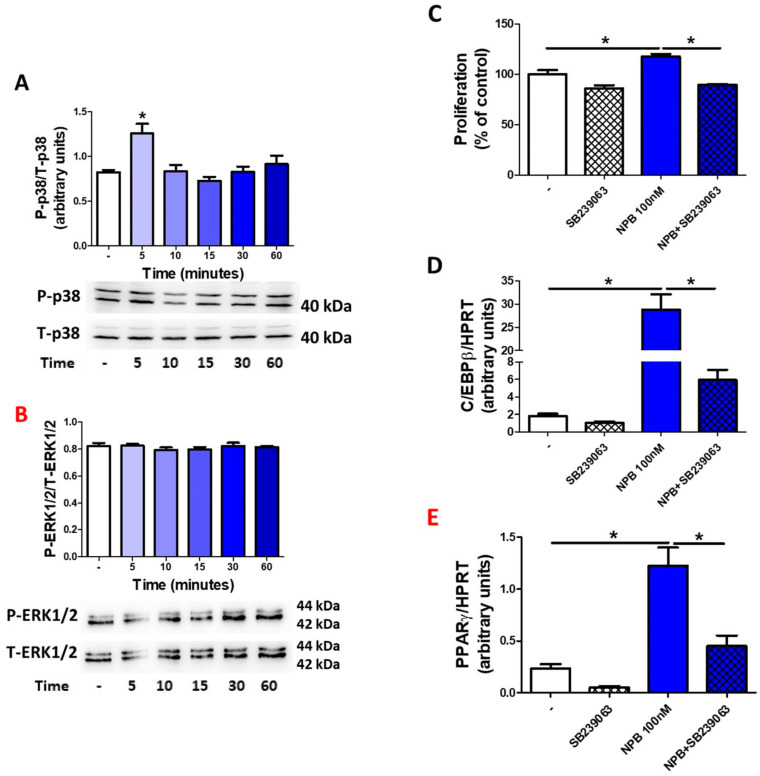
Detection and quantification of p38 (**A**) and ERK1/2 (**B**) phosphorylation in porcine preadipocytes exposed to NPB (100 nmol/L) for the indicated time points. Proliferation of preadipocytes exposed to NPB (100 nmol/l) in the presence or absence of SB239063 (5 μmol/L) for 24 h (**C**). Expression of C/EBPβ (**D**) and PPARγ (**E**) mRNA in porcine preadipocytes differentiated with or without NPB (100 nmol/L) in the presence or absence of SB239063 (5 μmol/L; 24 h). Results are the mean ± SEM, (n = 3–5). Statistical significance was considered when *p <* 0.05 (*).

As demonstrated in Figure 5A, NPB (100 nmol/L) stimulated the phosphorylation of p38 after 5 min. In contrast, NPB failed to modulate ERK1/2 phosphorylation in porcine preadipocytes (Figure 5B). Furthermore, NPB was not able to stimulate preadipocyte proliferation (Figure 5C) as well as C/EBPβ mRNA expression in the presence of SB239063 (a pharmacological blocker of p38 kinase [36]) (Figure 5D). Similarly, we observed the lack of NPB stimulated PPARγ mRNA expression increasing when the SB239063 blocker of p38 kinase was added to the medium during 24 h of differentiation culture (Figure 5E).

These results show that NPB modulates white adipogenesis in porcine preadipocytes via a p38-dependent mechanism.

## 3. Discussion

In this study, we demonstrated that NPB may be involved in white adipogenesis in porcine preadipocytes. Several lines of evidence support our findings. We identified NPB as well as both of its receptors in porcine preadipocytes. This observation is consistent with our previous study, in which we detected the expression of NPB as well as NPBWR1 in rodent white fat precursor cells (rat primary preadipocytes and 3T3-L1 cells) [27]. Surprisingly, in our current study, we found that protein production, but not the mRNA expression of NPBWR1 and NPBWR2, was downregulated during the differentiation process. It is possible that changes in the protein level of both NPB receptors resulted from changes in the translational and post-translational regulation of NPBWR1 and NPBWR2 protein production during adipogenesis.

Furthermore, we studied the effects of NPB on cell replication as well as the expression on transcription factors (C/EBPβ, C/EBPα and PPARγ), which play relevant roles in controlling the differentiation of white preadipocytes into mature fat cells [37]. Our results show that NPB stimulated the proliferation of preadipocytes. Interestingly, in our previous study, NPB failed to affect the proliferation of rat preadipocytes [27]. It cannot be excluded that these discrepancies resulted from the different species (rat vs. pig). Furthermore, in our rodent study, we utilized preadipocytes isolated from visceral epididymal fat depot, while in the present study, preadipocytes were obtained from dorsal subcutaneous adipose tissue samples. Importantly, it was demonstrated that in the adipose stem cells isolated from subcutaneous fat, the cell proliferation rate was significantly higher than in the cells derived from visceral adipose tissue [38]. It is also worth noting that visceral adipose tissue displays a higher differentiating capacity [39]. Furthermore, we found that NPB stimulated the expression of C/EBPβ, C/EBPα, and PPARγ. Consistently, we found that preadipocytes exposed to NPB showed a higher protein production of FABP4, which can be induced by PPARγ activation [40]. Nevertheless, it is important to note that the effects of NPB on the expression of these above-mentioned genes were observed in cells differentiated for one day only. Notably, in our previous study in rat preadipocytes, NPB was more effective in stimulating the differentiation process in cells differentiated for one or three days, in comparison with cells differentiated for one week [27]. Therefore, these data suggest that NPB may mainly be involved in early stages of the differentiation process. In this context, we observed higher levels of expression of NPB receptors in cells differentiated for one day compared with cells exposed to a differentiation medium for six days. Furthermore, it is also possible that prolonged exposition of cells to NPB may lead to desensitization of NPB receptors, in analogy to GPCR [41]. To further confirm the proadipogenic activity of NPB, we assessed its effect on lipid accumulation and lipid droplet formation in the preadipocytes during differentiation. Enhanced lipid accumulation and the formation of lipid droplets hallmark the differentiation of preadipocytes into mature adipocytes [15]. Indeed, NPB enhanced the lipid accumulation in preadipocytes during the differentiation process. Overall, these results provide evidence that NPB promotes the differentiation of porcine preadipocytes into mature fat cells.

In this study, we also characterized the potential mechanism by which NPB may stimulate adipogenesis in porcine fat precursor cells. Recently, we found that NPB is able to promote ERK1/2 phosphorylation in insulin-producing INS-1E cells [10]. Furthermore, we reported that NPB induces the phosphorylation of p38 kinase in rat white and brown primary preadipocytes [14,27]. As indicated in the introduction, both protein kinases, p-38 and ERK1/2, are involved in controlling adipogenesis [42,43,44], so we therefore studied whether NPB is able to modulate the phosphorylation of these protein kinases in porcine preadipocytes. Our results show that NPB induces p38 phosphorylation while it fails to change the phosphorylation level of ERK1/2 kinases. To further support our findings, we studied the effect of the pharmacological blockade of p38 by SB239063 on NPB-induced cell proliferation and the differentiation of porcine preadipocytes. NPB failed to promote cell proliferation in the presence of SB239063. Furthermore, in the presence of SB239063, NPB had no effect on the expression of C/EBPβ and PPARγ. Furthermore, it was found that in various cell types, p38 could induce the transcription and transcriptional activity of C/EBPβ [45,46,47], which is critically involved in early adipogenesis [15]. Overall, these results provide strong evidence that NPB stimulates adipogenesis in porcine preadipocytes in a p38-dependend manner. Nevertheless, it needs to be pointed out that studies on p38 in controlling adipogenesis provided contradictory results, and the role of p38 was extensively discussed in a review by Leivia et al. [44]. For example, it was demonstrated that the pharmacological blockade of p38 in 3T3-L1 cells suppresses adipogenesis [48]. Others have reported that the accelerated activation of p38 in 3T3-L1 cells may be relevant in the downregulation of adipogenesis [49].

To date, our knowledge regarding the role of p38 in adipogenesis in pigs is rather moderate. However, it is worth mentioning that the proadipogenic role of p38 is supported by the results of other studies showing that miR-29b/c downregulates adipogenesis in porcine preadipocytes, which was associated with the suppression of p38 phosphorylation [50]. Nevertheless, the role of p38 kinase in white fat adipogenesis requires more studies.

Our study had several limitations. Despite the utilization of primary preadipocytes, it needs to be pointed out that our experiments were performed only in vitro. Therefore, it remains to be investigated whether NPB modulates adipogenesis in pigs in vivo. Furthermore, NPB doses used in our study were relatively high, which is usually the case in vitro studies using isolated cells [51]. It was demonstrated that the collagenases used to isolate the primary cells may lead to the loss or reduction in cellular membrane receptors [52]. Therefore, higher doses of ligands are usually required for in vitro studies.

The physiological relevance of our findings remains to be confirmed in vivo. The identification of new peptide hormones importantly involved in fat tissue formation and its distribution would be useful for animal breeding programs aiming to improve the efficiency of porcine meat production.

## 4. Materials and Methods

### 4.1. Reagents

Porcine (6-Br)-neuropeptide B-29 aa was synthetized by Novazym (Novazym Life Science & Technology, Poznan, Poland). Culture media and fetal bovine serum (FBS) were purchased from Biowest (Nuaillé, France). The BrdU Cell Proliferation ELISA Kit was from Roche Diagnostics (Basel, Switzerland). Antibodies anti-phospho-p38 MAPK (Thr180/Tyr182) (#9211), p38 MAPK (#9212), phospho-ERK1/2 (#9101), ERK1/2 (#9102), PPARγ (#95128), C/EBPβ (#3082), FABP4 (#2120). Anti-rabbit IgG HRP-linked antibody (#7074) and anti-mouse IgG HRP-linked antibody (#7076) were from Cell Signaling Technology (Danvers, MA, USA). The anti-NPBWR1 antibody (bs-8618R) was from Bioss Antibodies (Wobur, MA, USA); anti-NPBWR2 (STJ192639) was purchased from St John’s Laboratory Ltd. (London, UK); anti-NPB antibody (LS-C150319) was from Lifespan Biosciences (Seattle, WA, USA). Anti-β-actin (A1978) was from Sigma-Aldrich (St. Louis, MO, USA). Other reagents were purchased from Sigma-Aldrich, unless otherwise stated.

### 4.2. Cell Isolation and Culture

The isolation of primary porcine preadipocytes was carried out according to a previously described technique [53,54] based on Ramsay at al. [54]. The Zlotnicka race piglets approx. one week old (weighing 7–10 kg) were purchased from an Experimental Station of the Poznan University of Life Sciences in Zlotniki and all procedures were conducted in accordance with the recommendations and ethical standards set by the National Ethics Commission for Investigations on Animals in Poland. In brief, preadipocyte-rich stromal-vascular cells were isolated from dorsal subcutaneous adipose tissue samples cut in small pieces and placed in Krebs-Ringer buffer supplemented with 3% BSA, 5 mM glucose, and antibiotics (100 U/mL penicillin and 0.1 mg/mL streptomycin). Fat pads were washed three times with Krebs Ringer buffer, cut into small pieces, and digested for 60 min in 37 °C water bath using collagenase type II (3 mg/mL, Sigma-Aldrich, St. Louis, MO, USA). The cell suspension was centrifuged (450× *g*, 10 min) and the supernatant containing mature adipocytes was discarded. Red Blood Cell Lysing buffer was added and after erythrocytes hemolysis, the cell suspension was mixed with 1:1 Krebs Ringer buffer followed by filtration through 100 μm, and then 40 μm meshes. After the final centrifugation (450× *g*, 10 min), cells were suspended in DMEM-F12 medium (10% FBS and antibiotics). Next, cell viability (>95%) was evaluated using 0.4% Trypan Blue dye and cells were counted with a Fűsch-Rosenthal counting chamber. Cells suspended in the culture medium were placed in standard conditions (37 °C incubator with 95% air and 5% CO_2_) in appropriate multi-well plates for 24 h pre-culture. Then, the cells were cultured to stimulate differentiation (one and six days) or used to study proliferation (24 and 48 h).

### 4.3. Cell Proliferation Assay

Cell proliferation was analyzed as described in our publication [53]. Preadipocytes (4 × 10^3^ cells/well of 96-wells plates) pre-cultured for 24 h were washed with warm PBS and DMEM/F12 serum-free medium containing antibiotics and 0.1% BSA (heat shocked fraction, protease free, fatty acid free, essentially globulin free, Sigma-Aldrich) was added. Cells were incubated in this medium in the presence or absence of NPB (1, 10, and 100 nmol/L) for 24 or 48 h. Cell proliferation was analyzed using a Cell Proliferation ELISA BrdU Colorimetric Kit (Roche Diagnostics, Basel, Switzerland) according to the manufacturer’s instructions. In brief, BrdU solution (10 µmol/L) was added and cells were incubated for 4 h. Next, the medium was discarded, cells were fixed (30 min at RT), and the anti-BrdU-POD conjugate was added. The incubation was conducted for 70 min at RT. Afterward, the cells were washed three times with PBS, the substrate solution (TMB) was added, and the plate was incubated for approximately 20 min. After stopping the reaction (25 µL of 1 M H_2_SO_4_), the absorbance was read using a Synergy 2 Multi-Mode Microplate Reader (BioTek, Winooski, VT, USA) at a 450 nm wavelength.

### 4.4. Differentiation of White Preadipocytes

The differentiation process was carried out as previously described [53]. In brief, after 24 h of preculture, the preadipocytes (approx. 80% of confluency) were washed with pre-warmed PBS and exposed to differentiation medium (DMEM/F1) supplemented with 850 nM insulin, 10 nM dexamethasone, 2 nM triiodothyronine, and antibiotics (10,000 IU/mL penicillin and 10,000 µg/mL streptomycin), and in the presence or absence of NPB (10–100 nmol/L). The differentiation medium was exchanged every two days. Incubations were carried out for one or six days. For protein extraction and RNA isolation, the cells were differentiated in 6-well plates while for intracellular lipid evaluation, on 12-well plates.

### 4.5. Oil Red O (ORO) Staining and Microphotographs

Briefly, cells were washed with warmed PBS, fixed with 10% formalin in PBS twice (10 min, 1 h at room temperature), and stained with ORO (working solution freshly prepared by mixing six parts of ORO stock solution containing 0.7 g/200 mL isopropanol with four parts of distilled water) for 10 min at RT. Next, the stained cells were washed four times with distilled water and photographed using LSM 510 inverted microscopy (Carl Zeiss, Oberkochen, Germany). Images were processed using Axio vision v. 4.6 software. For quantitation, cells were dried and ORO was eluted using 100% isopropanol. Absorbances were read using Synergy 2 Multi-Mode Microplate Reader (BioTek, Winooski, VT, USA) at a 520 nm wavelength.

### 4.6. Reverse Transcription and Real-Time qPCR

Total RNA was isolated using the Extrazol reagent (DNA-Gdańsk, Gdańsk, Poland) according to the manufacturer’s instruction. RNA quality and concentration were assessed spectrophotometrically (NanoDrop 1000, Thermo Fisher Scientific, Waltham, MA, USA). A total of 1 µg of total RNA was used to generate cDNA using the FIREScript RT cDNA Synthesis Mix (Solis BioDyne, Tartu, Estonia) with oligo (dT) and random primers. Obtained cDNA was diluted one to ten in nuclease-free water and the real-time quantitative PCR reactions containing HOT FIREPol EvaGreen qPCR Mix Plus (Solis BioDyne, Tatru, Estonia) were carried out (QuantStudio 12 K Flex, Thermo Fisher Scientific, Waltham, MA, USA). The sequences of primers and gene accession numbers are listed in Table 1. The relative quantification was calculated using the double delta CT method. The expressions of target genes were normalized to the HPRT mRNA expression levels.

### 4.7. Western Blot Immunodetection

Cells seeded on 6-well plates were used to obtain protein extracts using RIPA-lysing buffer (50 mM Tris-HCl, 150 mM NaCl, 0.25% deoxycholic acid, 1% Np-40, 1 mM EDTA, ph. 7.4) in the presence of protease and phosphatase inhibitor cocktail (Sigma-Aldrich). Protein concentration was measured using the BCA Protein Assay Kit (ThermoScientific, Rockford, IL, USA); 30 µg protein was resolved on 5–12/18% Tris-HCl SDS-PAGE gel and blotted onto an Immun-Blot PVDF membrane (Bio-Rad Laboratories, Munich, Germany), using Pierce Power Blot Cassette (Thermo Fisher Scientific). Membranes were blocked (5% BSA in Tris-buffered saline containing 0.1% Tween 20, TBST) for 1 h at RT and incubation with the primary antibody (1:1000 dilution) was carried out overnight at 4 °C. The next day, the membranes were washed with TBST (3 × 5 min) and incubated with the secondary anti-rabbit IgG HRP-linked goat antibody (1:5000 dilution, 1 h at RT). The chemiluminescence reaction was performed using Immobilon Forte Western HRP substrate (Merck Millipore, Burlington, MA, USA) on a ChemiDoc Touch Gel Imaging System (Bio-Rad, Hercules, CA, USA). Afterward, the membrane was stripped and incubated with antibodies against β-actin (1:5000 dilution), followed by the secondary anti-mouse IgG HRP-linked goat antibody (1:5000 dilution, 1 h at RT). Signal intensity was analyzed using Image Lab 6.1 software (Bio-Rad Laboratories, Munich, Germany).

### 4.8. Statistical Analysis Statistical Analysis

One way ANOVA followed by the Bonferroni post hoc test statistical analysis test was performed. To analyze differences between the two groups, the *t*-test was used. Results are shown as the means ± SEM and were derived from independently performed experiments repeated at least two times. Differences with a *p*-value less than or equal to 0.05 were considered statistically significant (*).

## 5. Conclusions

In conclusion, we report that NPB stimulates the proliferation and differentiation of porcine preadipocytes into adipocytes via the p38-dependent mechanism.

## Figures and Tables

**Table 1:** Sequences of the porcine PCR primers used in the study.

Gene	Left Primer (5′-3′)	Right Primer (5′-3′)	NCBI Reference Sequence
NPB NPBWR1 NPBWR2 C/EBPα C/EBPβ PPARγ HPRT	gatgtcttcctgtccctccg caacatagccgacttcctgc gacacaacatcaccttcccg ctcaccgctccgattcctac tccgatctcttctccgacga ttccatgctgtcatgggtgaaa cattcctatgactgtagattt	gaaaagacggggcttggtac agaggctggagaaggtgttg gcagatgagggagtacacca tccttctattgcgggggaga caggctcacgtagccgtatt accatggtcacctcttgtgaa ctttggattatgctgcttg	XM_021066433.1 XM_003355060.4 XM_021077700.1 XM_003127015.4 AB569088.1 XM_013981981.2 NM_001032376

## Data Availability

The data presented in this study are available on request from the corresponding author.

## Abbreviations

aP2: adipocyte protein 2; BAT, brown adipose tissue; BrdU, 5-bromo-2′-deoxyuridine; C/EBPα, CCAAT/enhancer-binding protein alpha; C/EBPβ, CCAAT/enhancer-binding protein beta; DMEM, Dulbecco’s modified Eagle’s medium; ERK 1/2, extracellular signal regulated kinases 1/2; FABP4, fatty acid binding protein 4; GLUT-4, glucose transporter type 4; GPCR, G protein-coupled receptor; Hprt, hypoxanthine-guanine phosphoribosyltransferase; MAPK, mitogen activated protein kinase; MCPIP, monocyte chemotactic Protein-1-induced protein; MSCs, mesenchymal stem cells; NPB, neuropeptide B; NPBWR1, neuropeptides B/W receptor 1; NPBWR2, neuropeptides B/W receptor 2; ORO, oil red O; PEPCK, phosphenolpyruvate carboxykinase; PPARγ, peroxisome proliferator-activated receptor gamma; RT, room temperature; SVF, stromal vascular fraction; T3, triiodothyronine; WAT, white adipose tissue.
